# The role of subclinical depressive symptomatology during the prenatal period in cortisol rhythm alterations and postpartum depression risk

**DOI:** 10.1192/j.eurpsy.2022.293

**Published:** 2022-09-01

**Authors:** Á. Castro Quintas, M. Daura-Corral, E. Eixarch, F. Crispi, L. De La Fuente Tomas, M. Rocavert Barranco, A. Miguel Valero, L. Marques Feixa, H. Palma Gudiel, M.P. Garcia-Portilla, L. Fañanas

**Affiliations:** 1 University of Barcelona, Evolutionary Biology, Ecology And Environmental Sciences, Barcelona, Spain; 2 Centre for Biomedical Research Network on Mental Health (CIBERSAM), Instituto De Salut Carlos Iii, Madrid, Spain; 3 Maternitat Hospital Clinic Barcelona, Bcnatal Fetal Medicine Research Center, Barcelona, Spain; 4 University of Oviedo, Department Of Psychiatry, Oviedo, Spain

## Abstract

**Introduction:**

Cortisol, the hormonal endpoint of Hypothalamic Pituitary Adrenal (HPA) axis, coordinates the body response in front of daily stressful situations. Disturbances in cortisol circadian rhythm have been implicated in the pathophysiology of depression and neurodevelopment lasting consequences. Although pregnancy entails a progressively increase in cortisol levels, the consequences of subclinical depression traits during pregnancy in cortisol circadian rhythm remains unclear.

**Objectives:**

To analyze the impact of prenatal subclinical depressive symptomatology in cortisol circadian rhythm through pregnancy and its relevance for postpartum depression risk.

**Methods:**

A cohort of 112 healthy pregnant women (Mean age±SD=32.32±4.37) of the general population was followed throughout their first pregnancy and first two months of postpartum period. Diurnal salivary cortisol curve (four measures) was obtained for every trimester; the Area Under the Curve with respect to the ground (AUCg) and with respect to the increase (AUCi) were used as measures of basal HPA axis functioning. Depressive symptomatology was assessed every pregnancy trimester and postpartum period following EPDS criteria. All the analyses were adjusted for maternal age, weight, ethnicity and socioeconomic status and sample collection’s time.

**Results:**

Prenatal subclinical depressive symptomatology (EPDS>10) was associated with a blunted cortisol rhythm during first trimester (F= 3.913,p=.011) but not during second (F=2.629, p=056) or third trimesters (F=.411,p=.724). Furthermore, a logistic regression model showed a positive association between Prenatal subclinical depressive symptomatology and the risk of postpartum depression (χ^2^=13.8, p<.001,OR=9.6; 95%CI 2.5–35.5).

**Conclusions:**

Women with subclinical depressive symptomatology in early pregnancy had alterations in cortisol circadian rhythmicity and a higher risk of postpartum depression.

**Disclosure:**

No significant relationships.

